# An adaptive Epithelial-Mesenchymal Transition Program Enables Basal Epithelial Cells to Bypass Stress-Induced Stasis and Contributes to Metaplastic Breast Cancer Progenitor State

**DOI:** 10.21203/rs.3.rs-4980285/v1

**Published:** 2024-09-27

**Authors:** Joseph A. Caruso, Thea D. Tlsty

**Affiliations:** University of California San Francisco; University of California San Francisco

**Keywords:** Human mammary epithelial cells, Basal, Myoepithelial, TGF-β Pathway, Epithelial-mesenchymal transition, Epigenetic Regulation, Metaplastic Breast Cancer

## Abstract

**Background:**

Human mammary epithelial cell (HMEC) cultures encounter a stress-associated barrier termed stasis, during which most cells adopt a senescence-like phenotype. From these cultures, rare variants emerge from the basal epithelial population, re-initiating growth. Variants exhibit pre-malignant properties, including an aberrant epigenetic program that enables continued proliferation and acquisition of genetic changes. Following oncogenic transformation, variants produce tumors that recapitulate the histopathological characteristics of metaplastic breast cancer (MBC), a rare subtype characterized by squamous and mesenchymal differentiation.

**Methods:**

Using the conventional serum-free HMEC culture system, we probed the capacity for phenotypic plasticity inherent to basal epithelial cell populations from human breast tissue as they navigated stasis and emerged as variant populations.

**Results:**

We observed robust activation of a TGF-β-dependent epithelial-mesenchymal transition (EMT) program in basal epithelial cells during stasis, followed by subsequent attenuation of this program in emerging variants. Inhibiting the TGF-β pathway or depleting the EMT regulators Snail or Slug allowed basal epithelial cells to collectively bypass stasis, demonstrating that cellular dysfunction and arrest resulting from TGF-β and EMT activation are central to this *in vitro* barrier. The spontaneous emergence of variants from stasis cultures was associated with a restricted EMT trajectory, which diverted cells away from a complete mesenchymal state characterized by irreversible growth arrest, and instead limited variants to epithelial and intermediate EMT states associated with greater proliferative capacity and stemness. Epigenetic mechanisms, which contributed to the dysregulated growth control characteristic of the variant phenotype, also contributed to the constrained EMT program in variants. By overcoming the cellular dysfunction and growth arrest resulting from TGF-β and EMT activation, variants exhibited increased oncogenic transformation efficiency compared to pre-stasis basal epithelial cells. Inhibiting the TGF-β pathway prior to stasis significantly reduced EMT in the basal epithelial population, alleviated selective pressure driving variant emergence, and enhanced oncogenic transformation efficiency, resulting in tumors with markedly diminished metaplastic differentiation.

**Conclusions:**

This study reveals how adaptive EMT reprogramming governs basal epithelial cell fate decisions and contributes to the development of MBC progenitors by restricting access to terminal mesenchymal states that induce growth arrest and, instead, favoring intermediate states with enhanced tumorigenic potential.

## Background

The milk-producing lobules and ductal networks within a healthy human breast consist of an inner luminal layer of polarized columnar epithelial cells and an outer basal layer of contractile myoepithelial cells. Both luminal and basal epithelia contain hierarchies of progenitor cells [1–6], whose transformation significantly contributes to the histological and molecular heterogeneity observed among breast cancers [7–12]. These cancers are commonly classified into subtypes that can express markers of luminal or basal differentiation states [13–15]. However, most, including those with characteristically basal expression patterns, are believed to originate from progenitor cell populations in the luminal epithelium [7, 11, 16]. Only rare metaplastic breast cancers (MBCs), representing 0.2–5 percent of all breast cancers [17], are proposed to originate from basal progenitor populations [9] primarily responsible for the maintenance of the myoepithelial layer [1, 2].

MBC is a histologically defined classification typified by differentiation into cell types that are not normally found in breast tissue, including squamous and mesenchymal (e.g., spindle cell, chondroid, and osseous metaplasia) phenotypes generating foci of bone, muscle and cartilage cells. These cancers predominantly fall within claudin-low or basal-like intrinsic molecular subtypes and are generally clinically classified as triple-negative breast cancers (TNBCs) [17, 18]. Epithelial-mesenchymal transition (EMT) is closely associated with the pathogenesis of MBC and is believed to contribute to its comparatively poor prognosis, chemoresistance, and high metastatic potential. EMT is a developmental process in which epithelial cells acquire mesenchymal characteristics, including increased motility and invasiveness, which can be reactivated in response to injurious or pathological conditions, including carcinogenesis [19, 20]. Notably, EMT is not a binary switch from the epithelial to mesenchymal state. Instead, it produces a spectrum of intermediate cellular states that exhibit both epithelial and mesenchymal characteristics. These intermediate states are associated with an increase in stem cell properties, tumorigenicity, drug resistance, and metastasis [21–28].

The human mammary epithelial cell (HMEC) culture system, developed more than 40 years ago [29] by the Stampfer group, remains one of the most widely used platforms for studying epithelial cell biology and carcinogenesis. Establishing HMEC cultures requires mincing healthy human breast tissue, enzymatically digesting matrix components, and removing stromal cell types [29, 30]. In healthy tissues, cellular cooperation relies on complex communication networks involving a vast array of biochemical and biophysical interactions, including interactions between parenchymal and stromal cells, the extracellular matrix, and the systemic milieu [31]. Constantly integrating information from these networks defines a cell’s operational state and sets limits on cellular behavior [32]. Indeed, after 5–20 population doublings in culture, HMECs enter stasis, a ‘stress-associated’ barrier that is dependent on the engagement of the tumor suppressor retinoblastoma (Rb) and characterized by most cells adopting a senescence-like phenotype [8, 33, 34]. Variants, exclusively derived from the basal compartment [9], evade this fate and initiate a second phase of logarithmic growth as clonal outgrowths from stasis cultures. These variant basal epithelial cells (vHMECs) continue to experience telomere erosion, allowing for an additional 20–70 population doublings before reaching a telomeric crisis state known as agonescence [29, 34]. Because of their extended lifespans, variants are more broadly available from commercial sources and are often marketed simply as HMECs. These cells have been immortalized and oncogenically transformed into cell lines that are widely used in laboratories worldwide to model breast epithelial biology and carcinogenesis. Thus, vHMECs have profoundly influenced many fields, including the study of stromal-epithelial interactions, aging, EMT, and stem cell biology.

Variant basal epithelial outgrowths that emerge from stasis and dominate post-selection HMEC cultures exhibit compromised growth control and acquire other characteristics associated with malignant phenotypes. Many of these alterations result from the activation of an epigenetic program, which is surprisingly consistent between donors [35–41]. Characteristic epigenetic alterations include hypermethylation of specific genes and loci targeted by polycomb repressor complex 2 (PRC2), including the promoters of genes regulating growth control and differentiation, such as cyclin-dependent kinase inhibitor 2A (CDKN2A) and homeobox A9 [35, 37, 41]. In addition to alterations in the chromatin landscape, cytogenetic analysis reveals an accumulation of chromosomal abnormalities in variant cultures, including translocations, deletions, telomeric associations, polyploidy, and aneuploidy [34]. Collectively, modifications in the variant HMEC state are sufficient to allow these cells to tolerate oncogenic events, such as the introduction of oncogenic forms of RAS, without undergoing oncogene-induced senescence [39]. Additional factors, such as the viral oncoprotein SV40 large T antigen and telomerase reverse transcriptase (TERT), are required to fully immortalize these cells, which is a prerequisite for the introduction of a transforming oncogene to achieve tumorigenicity in immunocompromised mice [42]. Notably, the malignant transformation of variant cultures characteristically results in the development of tumors that recapitulate the histopathological characteristics of the rare MBC subtype. Thus, many widely used breast cancer models derived from HMEC cultures, such as MCF-10AT, HMT-3522 T4–2, and HMLER, form tumors exhibiting metaplastic squamous and spindle cell differentiation [8, 42, 43].

In this study, we observed robust activation of the TGF-β pathway and EMT as HMEC cultures entered stasis, which was followed by the attenuation of these programs in emerging variant basal epithelial populations. In this system, activation of the TGF-β pathway and EMT was associated with cellular dysfunction, driving cells toward a senescent-like phenotype. Rare variants that spontaneously emerged from stasis cultures exhibited a restricted EMT trajectory, which prevented their complete transition to a mesenchymal state characterized by P-cadherin loss and irreversible growth arrest, instead confining them to dynamic transitions between epithelial and intermediate EMT states that maintained P-cadherin expression. The aberrant epigenetic program activated in variants not only compromised growth control and differentiation [35–41] but also contributed to the restricted EMT program observed in variants. The variant phenotype was associated with increased susceptibility to oncogenic transformation compared to unselected, pre-stasis basal epithelial cells and produced tumors with metaplastic elements. Pharmacological inhibition of the TGF-β pathway in cultured basal epithelial cells limited EMT program activation, reduced the selective pressures underlying variant emergence, and enhanced tumorigenic potential in pre-stasis basal epithelial populations, which produced tumors with dramatically reduced metaplastic differentiation. Our findings suggest that in a perturbed microenvironment, the adaptability of the EMT program, favoring intermediate states over complete mesenchymal differentiation, is crucial for directing basal epithelial cells toward a progenitor state that can progress to MBC.

## Methods

Reagents were obtained from Thermo Fisher Scientific unless otherwise stated.

### Tissue Dissociation

Disease-free breast tissue from women undergoing reduction mammoplasty was provided by the Cooperative Human Tissue Network (CHTN) Western Division, Nashville, TN, and Kaiser Foundation Research Institute, Oakland, CA. Human breast tissue was minced and enzymatically dissociated in RPMI with 2.1 mM L-glutamine and 25 mM HEPES supplemented with 10% (v/v) FBS (Atlanta Biologicals), 10 U/mL penicillin, 10 μg/mL streptomycin, 2.5 μg/mL Amphotericin B, 50 μg/mL gentamycin, Collagenase Type-2 (Worthington), and 100 U/mL hyaluronidase (Sigma-Aldrich) at 37°C for 12–16 hours. The digest was centrifuged at 300 g for 10 minutes and washed with RPMI-1640 supplemented with 10% FBS. Epithelial-enriched structures (referred to as tissue organoids) were recovered by filtration through a 150 μm nylon mesh filter and frozen for long-term storage in Dulbecco’s Modified Eagle Medium (DMEM) containing 50% (v/v) FBS and 10% (v/v) DMSO. The age and race of the donors are listed in Table S2.

Fluorescence-Activated Cell Sorting (FACS) and Flow Cytometry: Tissue organoids were dissociated into single cells using 5 U/mL Dispase (Stem Cell Technologies) containing 1 μg/mL DNAse I (Stem Cell Technologies) for 5–10 minutes and then 0.05% trypsin with 0.14 g/L Ethylenediaminetetraacetic Acid (EDTA) for 5–10 minutes. Cultured cells were collected from the tissue culture plates by incubating them for 10–15 minutes with TrypLE. The cells were washed with Phosphate-Buffered Saline (PBS) + 1% Bovine Serum Albumin (BSA), filtered through a 40-μm cell strainer (Falcon), resuspended at a concentration of 1 × 10^6^ cells in 100 μL of PBS + 1% BSA, and stained for 15 minutes at room temperature with the appropriate antibody combination. When necessary, the cells were washed with PBS + 1% BSA and stained for 15 minutes at room temperature with 1 μL streptavidin conjugated with BV785 (BioLegend) and 0.5 μg/mL DAPI (Sigma). The cells were washed, resuspended at 1 × 10^6^ cells in 100 μL PBS + 1% BSA, filtered through a 40-μm cell strainer, and loaded onto a FACS Aria II Cell Sorter (BD) fitted with a 100 μm nozzle. To isolate basal epithelial cells from tissue organoids, we immunostained cells with 5 μL of anti-CD10 mouse monoclonal antibody (mAb) (clone: HI10a) conjugated with PE (Biolegend), 1.5 μL of anti-Epcam mouse mAb (clone VU1D9) conjugated with FITC (Stem Cell Technologies), 4 μL of anti-CD49f rat mAb (clone GoH3) conjugated with APC (Biolegend), 8 μL of anti-CD2 mouse mAb (clone RPA-2.10) conjugated with Biotin (Becton Dickinson [BD]), 8 μL of anti-CD3 mouse mAb (clone HIT3a) conjugated with Biotin (BD), 8 μL of anti-CD16 mouse mAb (clone 3G8) conjugated with Biotin (BD), 8 μL of anti-CD64 mouse mAb (clone 10.1) conjugated with Biotin (BD), 4 μL of anti-CD31 mouse mAb (clone MBC78.2) conjugated with Biotin (ThermoFisher Scientific), and 1 μL of anti-CD45 mouse mAb (clone HI30) conjugated with Biotin (Biolegend). To isolate EMT phenotypes from cultured basal epithelial cells, we used 15 μL of anti-P-cadherin mouse mAb (clone CSTEM29) conjugated with APC (Thermo Fisher Scientific) and 4 μL of anti-N-cadherin mouse mAb (clone 8C11) conjugated with PE (BioLegend). After gating out debris, doublets, dead cells (DAPI+), and, if applicable, unwanted cell types (BV785+), the remaining target populations expressing the desired immunoprofiles were collected into a 1.5 mL tube containing 0.5 mL cell culture medium. The HMECs were pulsed with 10 μM EdU for one hour before the proliferation index was determined. The Click-iT Plus EdU Alexa Fluor 488 Flow Cytometry Assay Kit and FxCycle PI/RNase Staining Solution (Thermo Fisher Scientific) were used according to the manufacturer’s instructions. The cells were analyzed using an LSRFortessa flow cytometer (BD Biosciences).

### Cell Culture

Dissociated human breast tissue organoids were plated in T-75 flasks (Corning) in Mammary Epithelial Cell Growth Medium (MEGM, Lonza), which includes hydrocortisone, human epidermal growth factor, insulin, bovine pituitary extract (BPE), gentamicin, and amphotericin B. After the initial plating, differential trypsinization was performed to selectively detach and aspirate unwanted fibroblasts, followed by collection of epithelial cells for further passaging. Sorted populations were cultured in one well of a six-well plate, transferred to a p100 plate, and then to a T-75 flask. Subculturing was performed at 70–80% confluence using TrypLE Express for cell dissociation. Cells were incubated at 37°C in a humidified atmosphere containing 5% CO^2^. The medium was changed every two to three days. Cell viability and counts were assessed using trypan blue exclusion and a Countess automated cell counter (Thermo Fisher Scientific) before reseeding or experimental use. The number of population doubling (PD) per passage was determined using the equation PD = log [A/B]/log2, where A is the number of collected cells and B is the number of plated cells. Attachment efficiency was consistently greater than 95% during the exponential growth phase. Cell populations were frozen in cryopreservation medium composed of 90% FBS and 10% DMSO (Sigma-Aldrich), aliquoted into cryovials, and gradually cooled to −80°C before being transferred to liquid nitrogen for long-term storage. The TGF-β inhibitor A83–01 (Tocris) was dissolved in DMSO at 1 mM and used at a concentration of 0.5 μM; an equivalent amount of DMSO was added to control cultures.

### Mammosphere Culture

Single cells were directly sorted into 24-well ultra-low attachment plates (Corning) at a density of 10,000 cells per well in serum-free mammary epithelial basal medium (MEBM) (Lonza) supplemented with B27 (Thermo Fisher Scientific), 20 ng/mL Epidermal Growth Factor (EGF, Peprotech), 20 ng/mL Fibroblast Growth Factor 2 (FGF2, Peprotech), and 4 μg/mL heparin (Stem Cell Technologies). Mammospheres were counted and collected by centrifugation at 100 × g after 10 days of culture, dissociated enzymatically for 5–10 minutes in TrypLE Express, filtered through a 40-μm cell strainer, and cultured again in suspension [44].

Lentiviral Transduction: Basal epithelial cell populations, 1 × 10^6^ in a single well of a six-well plate, were incubated with concentrated lentiviral particles at a multiplicity of infection of three with 8 μg/mL polybrene overnight for 12–16 hours. For transformation, the cells were infected sequentially with (1) TERT with a hygromycin selection marker (Genecopoeia), (2) SV40 small T and large T antigens with a puromycin selection marker (Genecopoeia), and (3) KRAS G12V with a blasticidin selection marker (Amsbio). Cells were selected using 200 μg/mL hygromycin (Sigma-Aldrich), 1 μg/mL puromycin (InvivoGen), and 10 μg/mL blasticidin (InvivoGen). Transformed cells were grown in DMEM/F12 (1:1) supplemented with 5% Newborn Calf Serum (Hyclone), 10 μg/mL insulin, 10 ng/mL EGF, and 1 μg/mL hydrocortisone. CRISPR-mediated knockout of core EMT transcription factors, Snail (SNAI1) and Slug (SNAI2), Zeb (ZEB1), and Twist (TWIST1) was carried out using transEDIT CRISPR gRNA target gene sets from Transomics. Three constructs were tested for each gene. To suppress the EMT program, we chose to continue with: SNAI1 TEVH-1091413-pCLIP-All-EFS-ZsGreen, SNAI2 TEVH-1091323-pCLIP-All-EFS-ZsGreen, and non-targeting control TELA1013-CRISPR-NT#1-pCLIP-All-EFS-ZsGreen. CRISPR-mediated knockout of EZH2 was carried out using the Edit-R Human hEF1a-EGFP All-in-one Lentiviral sgRNA construct (Horizon Discovery Biosciences): EZH2 VSGH12180–256507490 and non-targeting VSGC12063.

### Soft-Agar Assay

A solution of 1% agar (Sigma-Aldrich) in PBS was prepared, and 1.5 mL was added to each well of a six-well plate and allowed to gel at room temperature for 30 minutes as the bottom layer. Cells were prepared by suspending 1 × 10^6^ cells in 3 mL pre-warmed DMEM/F12 containing 20% (v/v) FBS, 10 U/mL penicillin, and 10 μg/mL streptomycin. The cell suspension was gently mixed with 2 mL of 1% agar in PBS to achieve a cell density of 20,000 cells/mL in 0.4% agar; 1 mL of this mixture was added to each well of the six-well plate and kept at 4°C for 10 minutes to allow quick gelling, followed by adding 0.5 mL culture medium on top of the agar gel. The cells were incubated at 37°C with 5% CO^2^, and the medium was changed every four days for four weeks. Colonies were stained with 0.5 mL of 0.005% Crystal Violet (Sigma-Aldrich) in PBS containing 4% formaldehyde for one hour and then rinsed with distilled water before counting the visible colonies.

### Xenograft Study

Exponentially growing cultures of oncogenically transformed cells (4 × 10^6^) were injected subcutaneously into 8–12-week-old NSG female mice in 50% Matrigel. Tumors were allowed to grow for 3–6 months. After the study, xenograft tumors were weighed, formalin-fixed, and paraffin-embedded at the UCSF Histology & Biomarker Core. The maximum tumor burden was not reached in any of the mice in this study.

### Sandwich ELISA

Analysis of conditioned media from cultured HMECs from three donors at P2 (pre-stasis), P4 (stasis), and P8 (post-stasis) was initially performed using the Raybiotech Quantibody Human Kiloplex array, which detects 1,000 human biomarkers, as a service by the manufacturer. We followed up on interesting candidates using sandwich ELISA kits from Raybiotech to detect TGF-β, Activin A, Serpin E1, Wnt4, R-Spondin2, DKK1, and Follistatin according to the manufacturer’s protocols. For these assays, conditioned media were generated by culturing 300,000 cells per well in a six-well plate in 1 mL of serum-free basal media for 24 h. Each analyte was measured in eight biological and three technical replicates. The concentration of each factor in the conditioned media was extrapolated from the corresponding standard curves.

Immunofluorescence: Cells were cultured on four-well glass chamber slides (Millipore). The cells were fixed with 4% paraformaldehyde in PBS for 15 minutes at room temperature and permeabilized for 10 minutes in PBS containing 0.25% Triton X-100. Non-specific binding was blocked with 10% donkey serum (Jackson Immunoresearch), 0.3M glycine, and 0.1% Tween-20 in PBS for one hour. Primary antibodies were applied overnight in 1% donkey serum and 0.1% Tween-20 in PBS. The following primary antibodies were used: p16 (E6H4, Roche) mouse mAb (pre-diluted), p21 Waf1/Cip1 (12D1, Cell Signaling Technologies) rabbit mAb (1:400), p63-α (D2K8X, Cell Signaling Technologies) XP rabbit mAb (1:200), α-tubulin (DM1A, Sigma-Aldrich) mouse mAb (1:4000), MUC1 (HMFG2, Abcam) mouse mAb (1:200), Lamin A + Lamin C (EPR4100, Abcam) rabbit mAb (1:500), cytokeratin 19 (EPR1579Y, Abcam), rabbit mAb (1:400), and cytokeratin 14 (LL002, Abcam) rabbit mAb (1:200). Alexa Fluor 555-conjugated phalloidin (1:400) was used to stain F-actin (Thermo Fisher Scientific). Secondary donkey anti-mouse 488 and anti-rabbit 555 antibodies (diluted 1:500) were added to 1% donkey serum and 0.1% Tween-20 in PBS for one hour. DAPI (0.2 μg/mL in PBS) was added for five minutes at room temperature. The slides were washed three times for three minutes between each step in PBS containing 0.1% Tween-20. The coverslips were mounted with Vectashield HardSet Mounting Medium (Vector Laboratories). Images were acquired using a Leica SP8 laser scanning confocal microscope (only Tubulin/F-actin and Lamin A/C) at a resolution of 1,024 × 1,024, processed using LASX software (Leica Microsystems) or a BZ-X800 fluorescence microscope (Keyence), and processed using ImageJ (version 2.14.0/1.54f).

Quantitative Polymerase Chain Reaction (qPCR): Total RNA was isolated from cells and treated with DNase I using the RNeasy Mini Kit (Qiagen). cDNA synthesis was performed using the High-Capacity cDNA Reverse Transcription Kit (Thermo Fisher Scientific). Quantitative PCR was performed on a CFX-96 (Bio-Rad Laboratories) thermocycler using 2x SsoFast Master Mix (Bio-Rad Laboratories) and analyzed using the standard curve method. A standard curve was prepared using the cDNA produced from Human Reference RNA (Agilent Technologies). Pre-made TaqMan Gene Expression Assays (Thermo Fisher Scientific) were used: ZEB1: Hs00232783_m1, SNAI1: Hs00195591_m1, SNAI2: Hs00161904_m1, TWIST1: Hs01675818_s1, CDH3: Hs00999915_m1, VIM: Hs00958111_m1, CDH1: Hs01023895_m1, CDH2: Hs00983056_m1, CDKN1A: Hs00355782_m1, MMP-2: Hs01548727_m1, FN1: Hs01549976_m1, CHRDL2: Hs01060234_m1, GREM1: Hs01879841_s1, and WNT5a: Hs00998537_m1. Custom primer-probe sets were used for GUSB: Forward Primer: CTCATTTGGAATTTTGCCGATT, Reverse Primer: CCGAGGAAGATCCCCTTTTTA, and Probe: FAM-TGAACAGTCACCGACGAGAGTGCTGGTA-TAM and CDKN2A (specific for p16^INK4A^ and not p15^ARF^): Forward Primer: CCAACGCACCGAATAGTTACG, Reverse Primer: GAGTGGCGGAGCTGCT, and Probe: FAM-CCGATCCAGGTCATGATG-TAM produced by Integrated DNA Technologies. GUSB expression was used to normalize variance in the input cDNA.

Western Blotting: Cells were washed with PBS and lysed using radioimmunoprecipitation assay (RIPA) buffer containing the HALT protease and phosphatase inhibitor cocktail. For each sample, a cell extract (corresponding to 50 μg of protein as determined by a Micro BCA Protein Assay Kit) was prepared in NuPAGE LDS Sample Buffer (4X) with Reducing Agent (10X), heated at 95°C for 15 minutes, and electrophoresed in each lane of a NuPAGE 4–12% Bis-Tris gel along with a BenchMark Pre-stained Protein Ladder (Thermo Fisher Scientific). Proteins were transferred onto a nitrocellulose membrane (Biorad) overnight at 4°C at 36 mV. The membrane was washed in TBS-T (0.05 M Tris-HCl pH 7.5, 0.15 M NaCl, 0.05% Tween-20), blocked for one hour at room temperature in blotto (5% nonfat dry milk in TBS-T), incubated with primary antibodies overnight at 4°C, washed in TBS-T, incubated with goat anti-rabbit horseradish peroxidase conjugate secondary antibody (Jackson Immunoresearch) 1:5000 in Blotto for one hour, washed in TBS-T, and developed with ECL Western Blotting Substrate. Chemiluminescent signals were detected by exposing the CL-XPosure Film to the membranes or using the KwikQuant Digital Western Blot Detection System. Primary antibodies used at a 1:1000 dilution from Cell Signaling Technologies were Slug (C19G7) rabbit mAb, ZEB1 (D80D3) rabbit mAb, Slug (C19G7) rabbit mAb, E-cadherin (24E10) rabbit mAb, N-cadherin (D4R1H) XP rabbit mAb, Vimentin (D21H3) XP rabbit mAb, RAS rabbit pAb, EZH2 (D2C9) XP rabbit mAb, Histone H3 (D1H2) XP rabbit mAb, and β-Actin (13E5) rabbit mAb; and from Active Motif Histone H3K27me3 (MABI 0323) mouse mAb. Full images of the western blots are shown in Supplemental Figs. S4 and S5.

Multiplex Immunohistochemistry: Sections (5 μm thick) were cut from FFPE tissue blocks and placed on positively charged Superfrost Plus microscopy slides. Slides were baked at 60°C overnight, deparaffinized in xylene, and rehydrated in graded ethanol (100%, 100%, 95%, 85%, and 70%) in distilled H O. Endogenous peroxidases were quenched with 3% H O (Sigma-Aldrich) diluted in phosphate-buffered saline (PBS). Heat-induced antigen retrieval was performed in citrate buffer pH 6.0 (Sigma-Aldrich) at 95°C for 15 minutes. Non-specific antibody binding was blocked using a Background Sniper (Biocare Medical). The tissue sections were incubated for one hour at room temperature (RT) with each primary antibody in 1% BSA and 30 minutes in pre-diluted MACH 2 conjugated anti-mouse or anti-rabbit secondary antibodies (Biocare Medical). The slides were washed in TNT buffer (0.1 M TRIS-HCL pH 7.5, 0.15 M NaCl, and 0.05% Tween-20) following blocking and three times for five minutes each after applying both primary and secondary antibodies. The signal was developed using a Tyramide Signal Amplification (TSA) solution (Akoya): FITC (two minutes), Cy3 (three minutes), or Cy5 (seven minutes). The antibody complex was removed by heating at 95°C in citrate buffer pH 6.0 for five minutes to allow for multiplex staining. Nuclei were counterstained with 3 μM DAPI in PBS for five minutes, washed in distilled H O, and mounted using Vectashield HardSet Mounting Medium (Vector Laboratories). The primary antibodies used from Cell Signaling Technologies were Slug (C19G7) rabbit mAb (1:1000), Snail (C15D3) rabbit mAb (1:1000), p63-α (D2K8X) XP rabbit mAb (1:2000), and vimentin (D21H3) rabbit mAb (1:5000); from AbCam Cytokeratin 5 (SP27) rabbit mAb (1:4000), Lamin A + Lamin C (EPR4100) rabbit mAb (1:10000), Cytokeratin 13 (EPR3671) rabbit mAb (1:2000), Ki67 (SP6) rabbit mAb (1:6000), and Cytokeratin 14 (SP53) rabbit mAb (1:3000).The slides were imaged using a BZ-X800 analyzer. Images were prepared for analysis using the ImageJ software (version 2.14.0/1.54f). Analysis was performed using the Qupath software (v0.5.0)[45]. Using the pre-trained models, nuclear segmentation was performed using StarDist[46], normalizePercentiles = 1, 99, threshold = 0.5, pixel size = 0.5, and cell expansion = 5.0. Individual classifiers were trained for each marker and combined to create a composite classifier for scoring the cells within the annotated regions.

### Statistical Analysis

For each graph, the mean and standard deviation are shown in red. Unless otherwise noted in the figure legend, each black circle indicates a single donor. Pairwise comparisons were performed using an unpaired Welch’s t-test (GraphPad Prism Software). Levels of significance used were *0.05 to 0.01, **0.01 to 0.001, ***0.001 to 0.0001, and ***<0.0001.

## Results

### Robust Activation of the TGF-β Pathway and Induction of EMT in Stasis HMECs Precede Variant Emergence

**Figure 1:**
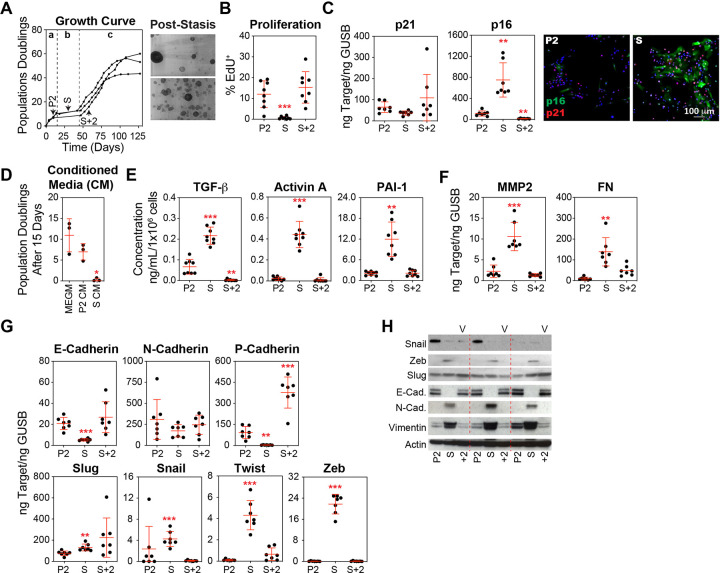
Transient Activation of EMT in Stasis HMECs Precedes Variant Emergence. **(A)** Human breast tissue samples from three donors were used to generate HMEC cultures in MEGM. The first passage after partial trypsinization was considered passage 1 (P1). At each passage, the cells were counted, and the cumulative population doubling was calculated. The resulting growth curves illustrate three distinct phases: the initial logarithmic growth phase (a), the stasis growth plateau (b), and the second logarithmic growth phase initiated by the expansion of variants (c). Protein, RNA, and conditioned media (CM) were collected from passage 2 (P2) HMECs, stasis HMECs (S), and variant HMECs at two passages beyond stasis (S+2). Crystal violet-stained flasks depict the pattern of variant emergence from the stasis cultures. **(B)** Cell proliferation was measured in HMEC cultures using the Click-iT EdU cell proliferation assay and quantified by flow cytometry using cells collected from P2, S, and S+2 cultures, which were re-plated overnight and pulsed with 10 μM EdU for one hour. **(C)** Gene expression analysis of p21 and p16 was performed on cDNA produced from cells collected from P2, S, and S+2 cultures by qPCR. Representative images of P2 and S HMECs immunostained with antibodies against p16 and p21, and counterstained with DAPI are shown. **(D)** CM collected directly from P2 and S HMEC cultures (incubated with cells for 24 hours) was mixed with an equal amount of fresh MEGM and used to culture freshly isolated HMECs (P1). **(E)**Sandwich ELISA kits were used to determine concentrations of TGF-β, Activin A, and PAI-1. An equal number of cells was harvested from P2, S, and S+2 cultures and cultured in basal media (supplements excluded) for 24 hours. Concentrations were normalized to the final cell number. **(F)** MMP-2 and FN were analyzed by qPCR. **(G)** E-cadherin, N-cadherin, P-cadherin, Slug, Snail, Twist, and Zeb were analyzed by qPCR. **(H)** Western blot analysis of lysates from P2, S, and S+2 cells using antibodies against Snail, Zeb, Slug, E-cadherin (E-Cad.), N-cadherin (N-Cad.), Vimentin, and Actin (loading control).

When cultured in serum-free Mammary Epithelial Cell Growth Medium (MEGM), HMECs isolated from three tissue donors experienced growth cessation after 5–10 population doublings. Stasis lasted between 20 and 40 days before the emergence of clonal variant populations, which re-initiated logarithmic growth (Fig. 1A). Nearly complete proliferative arrest was observed at stasis (Fig. 1B), marked by the upregulation of p16 and development of a senescence-like morphology (Fig. 1C). These results are consistent with previously published studies that have shown the clonal emergence of rare variants with pre-malignant properties within a field of growth-arrested, senescent-like epithelial cells [29, 33, 34, 41, 47, 48].

Previous studies of this culture system have primarily focused on comparing the initial pre-selection HMEC population to post-selection variants. In contrast, we chose to characterize stasis cultures, specifically focusing on the factors that enforce growth arrest. Conditioned media (CM) from stasis cultures, diluted 1:1 with fresh MEGM, induced significant growth arrest in early passage HMECs compared to fresh MEGM or CM from pre-stasis HMECs at passage 2 (P2), mixed 1:1 with fresh MEGM (Fig. 1D). Senescent and DNA double-strand breaks are associated with complex secretory programs, termed senescence-associated secretory phenotype (SASP) and stress-elicited extrinsic phenotype (SEEP), respectively, which can initiate or reinforce growth arrest in both autocrine and paracrine manners [49–51]. To characterize alterations in the cellular secretory program during HMEC culture, we collected CM and extracted cellular RNA and protein from cultures of pre-stasis HMECs at P2, growth-arrested stasis HMECs (S), and variant HMECs at two passages beyond stasis (S + 2). In stasis cultures, quantitative proteomic analysis using an antibody array (Table S1) indicated increased levels of transforming growth factor-beta (TGF-β) and Activin A, a member of the TGF-β superfamily. ELISA assays confirmed higher levels of TGF-β, Activin A, and Plasminogen Activator Inhibitor-1 (PAI-1), a biomarker of TGF-β pathway activation, in stasis cultures than in proliferating pre-stasis or variant HMEC cultures (Fig. 1E). Additionally, we detected an increase in matrix metalloproteinase 2 (MMP-2) and fibronectin (FN) transcript levels during stasis (Fig. 1F), which can be upregulated in response to the activation of the TGF-β pathway [52, 53]. Several cytokines and chemokines typical of SASP (IL-6, IL-8) [51] remained unchanged in the stasis cultures (Fig. S1A).

In stasis HMECs, we observed significant changes in gene expression indicative of EMT activation, which is consistent with the fundamental role of the TGF-β pathway in this process [54]. At the transcript level, we observed decreased levels of the epithelial markers E-cadherin (CDH1, concentrated in luminal epithelial cells) and P-cadherin (CDH3, typically expressed by basal epithelial cells), and no change in N-cadherin (CDH2, typically expressed by mesenchymal cells) in stasis HMECs compared to pre-stasis HMECs (Fig. 1G). We also observed increased levels of core EMT transcription factors Slug (SNAI2), Snail (SNAI1), Twist (TWIST1), and Zeb (ZEB1). Transcript levels of E-cadherin, Snail, Twist, and Zeb decreased in variant cultures compared to those in stasis cultures and were not significantly different from those observed in pre-stasis HMECs (Fig. 1G). In contrast, Slug transcript levels were increased in stasis HMECs and maintained in the variants. The derivation of variants from the basal compartment [9] likely accounts for the increased levels of P-cadherin observed in variant cultures compared with both pre-stasis and stasis cultures (Fig. 1G). Western blot analyses were consistent with the increased EMT activation, particularly during stasis. We observed decreased E-cadherin expression and increased Snail, Zeb, N-cadherin, and Vimentin expressions. Notably, protein expression was inconsistent with the changes in certain transcript levels (N-cadherin, Snail, and Slug), suggesting regulation by post-transcriptional processes commonly observed in EMT (Fig. 1H). In addition to TGF-β (Fig. 1D), several paracrine and autocrine factors involved in EMT [54] were robustly increased in stasis HMECs compared with those in either of the two active growth phases (Fig. S1B and S1C).

In summary, our initial observations demonstrated transient activation of the TGF-β pathway and engagement of EMT programs in cultured HMECs as they entered stasis. Remarkably, the emergence of variants from these cultures was associated with the attenuation of the TGF-β and EMT pathways.

#### Basal Epithelial Cells Exhibit Enhanced EMT Activation Compared to Luminal Epithelial Cells

**Figure 2:**
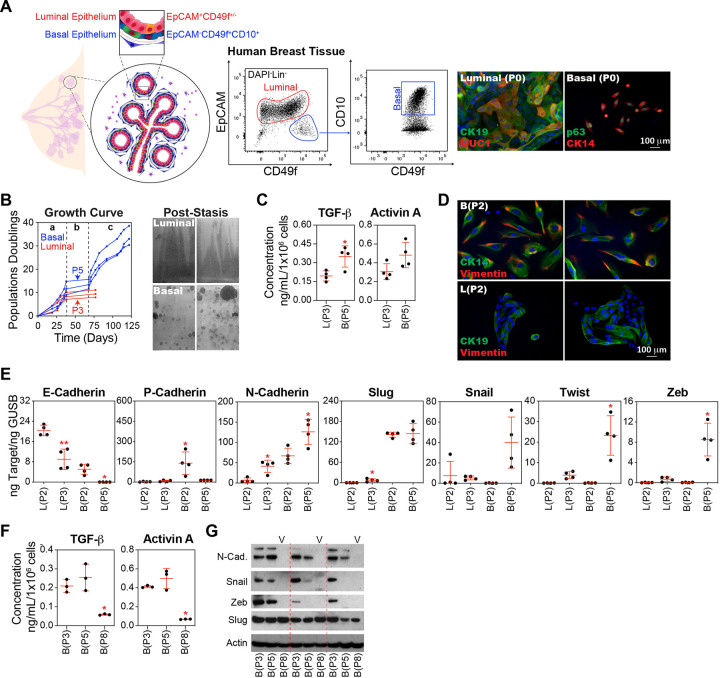
Basal Epithelial Cells Exhibit Enhanced TGF-β Signaling and EMT Activation Compared to Luminal Epithelial Cells. **(A)** Human breast tissues from the same three donors utilized in Fig. 1A were further processed into single cells. The cell suspension was stained with conjugated antibodies against CD10, EpCAM, CD49f, CD2, CD3, CD16, CD64, CD31, and CD45 then DAPI and conjugated streptavidin. Dead cells (DAPI^+^), doublets, and immune/stromal cell types (CD2^+^, CD3^+^, CD16^+^, CD64^+^, CD31^+^, and/or CD45^+^) were removed. The breast epithelium was separated into luminal (EpCAM^+^) and basal (EpCAM^−^ CD49f^+^CD10^+^) enriched populations by FACS and expanded separately in MEGM in 6-well plates. Representative wells were immunostained prior to passaging (P0) with the luminal markers cytokeratin 19 (CK19) and mucin 1 (MUC1) or the basal markers p63 and cytokeratin 14 (CK14). **(B)** Basal and luminal epithelial cells were further expanded in MEGM, cells were counted at each passage, and the growth curve of the cumulative population doubling over time was plotted. Cells and CM were collected from basal epithelial cells at passages B(P2), B(P5), and B(P7) and from luminal cells at passages L(P2) and L(P3). Crystal violet-stained flasks depict the pattern of variant cell emergence from basal epithelial cell cultures after stasis. **(C)** Sandwich ELISA kits were used to determine the TGF-β and Activin A concentrations. An equal number of cells was harvested from the B(P2), B(P5), L(P2), and L(P3) cultures and then cultured in basal media (supplements excluded) for 24 h. Concentrations were normalized to the final cell number. **(D)** Representative images of P2 basal and luminal epithelial cells immunostained with antibodies against pan-cytokeratin and vimentin and then counterstained with DAPI. **(E)** E-cadherin, P-cadherin, N-cadherin, Slug, Snail, Twist, and Zeb were analyzed by qPCR using cDNA produced from B(P2), B(P5), L(P2), and L(P3) cells. **(F)** Same as C, using basal epithelial cells from the B(P3), B(P5), and B(P8) cultures. **(G)** Western blot analysis of lysates from B(P2), B(P5), and B(P7) cultures, using antibodies against N-cadherin (N-Cad.), Snail, Zeb, Slug, and Actin (as loading controls).

The human breast epithelium is composed of two distinct layers: an inner luminal layer of polarized columnar epithelial cells and an outer basal layer of contractile myoepithelial cells. Both layers comprise a heterogeneous mixture of mature cells and cells at various stages of differentiation, including stem and progenitor cells [1–6]. For simplicity, we will refer to these complex populations as ‘luminal’ and ‘basal’ cells, acknowledging that these terms encompass a range of cellular states and phenotypes. To reduce the confounding variable of comparing a mixture of luminal and basal derivatives in pre-stasis cultures with exclusive basal derivatives in variant cultures [9], we used fluorescence-activated cell sorting (FACS) to separate basal epithelial cells (EPCAM^−^CD49f^+^CD10^+^) from luminal epithelial cells (EPCAM^+^) (Fig. 2A). When cultured in serum-free MEGM, the basal-enriched population from the three tissue donors entered stasis between 9 and 15 population doublings. These cultures consistently produced variant outgrowths 30 days after the cessation of growth at P5 (passage 5). In contrast, luminal cultures proliferated between 8 and 11 population doublings before growth arrest at P3, and did not produce variant colonies (Fig. 2B), consistent with a previous report [9]. Basal epithelial cells contributed significantly greater levels of TGF-β but not Activin A compared to luminal epithelial cells when analyzed at stasis, P3 for isolated luminal cells, and P5 for isolated basal epithelial cells (Fig. 2C). As early as P2, even before entering stasis, basal-enriched populations demonstrated a more dispersed and migratory phenotype and incorporated vimentin, a canonical marker of the mesenchymal phenotype, into their cytoskeleton, whereas luminal cells maintained tight colony morphologies and did not express significant levels of vimentin (Fig. 2D). At the transcriptional level, the basal epithelial population demonstrated a robust EMT response, as evidenced by significant increases in Twist, Zeb, and N-cadherin, and decreases in E-cadherin and P-cadherin between P2 and P5. Luminal epithelial cells demonstrated a significant increase in Slug and N-cadherin expression, and a decrease in E-cadherin expression (Fig. 2E). Thus, both luminal and basal epithelial cells demonstrated evidence of EMT; however, the magnitude of EMT-related changes in gene expression was markedly higher in the basal epithelial cells.

Similar to our observations in bulk HMECs, basal epithelial cell cultures were characterized by high levels of TGF-β and Activin A at early passages (P3 and P5), followed by a significant reduction in expression of these cytokines at later passages (P8) following the emergence of variants (Fig. 2F). Western blot analysis revealed high levels of N-cadherin, Snail, and Zeb in early passage (P3) and stasis (P5) basal epithelial cells, indicating activation of EMT. However, these components of the EMT program were downregulated in the variant cultures (P8). Notably, among the EMT transcription factors analyzed, Slug levels were consistently high across all passages, including the variants (Fig. 2G). A role of Slug in maintaining basal epithelial gene expression has been observed previously [55]. In summary, only the basal epithelial population contains variants [9]. This population recapitulated the transient activation of TGF-β and EMT phenotypes observed in bulk HMEC cultures, leading us to focus further on the basal epithelial cell population

#### Inhibition of TGF-β Signaling and EMT Transcription Factors Prevents Stasis in Basal Epithelial Cells

**Figure 3:**
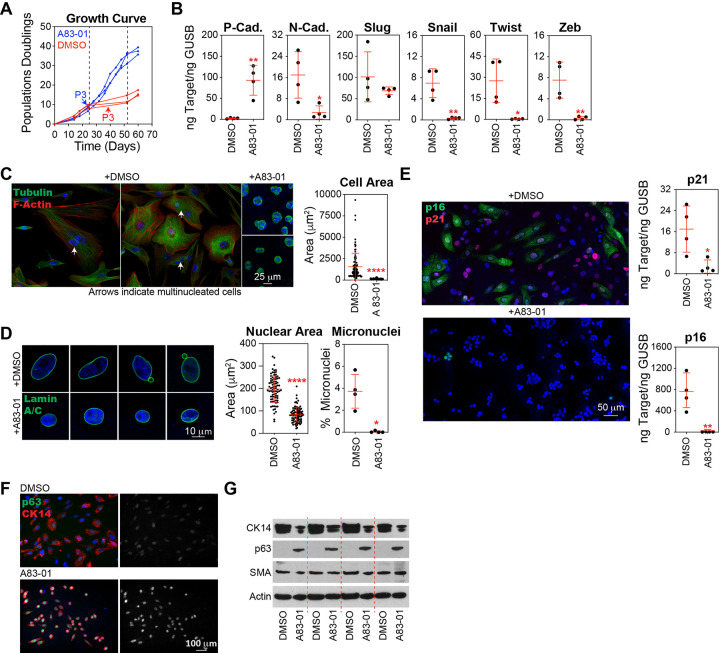
Inhibition of the TGF-β Pathway Prevents Stasis in Basal Epithelial Cells. **(A)** Basal epithelial cells, generated as introduced in Fig. 2A, were expanded in MEGM with 500 nM A83–01 or DMSO, the cells were counted, and the growth curve of the cumulative population doubling over time was plotted. Cells and CM were collected at P3, as indicated by arrows. **(B)**P-cadherin, N-cadherin, Slug, Snail, Twist, and Zeb were analyzed by qPCR using cDNA produced from basal epithelial cells at P3 treated with A83–01 or DMSO. **(C)**Representative images of P3 basal epithelial cells treated with A83–01 or DMSO immunostained with an antibody against β-tubulin and phalloidin, which stain F-actin, and counterstained with DAPI. The cells were segmented using ImageJ software, and the cell size was calculated. Data from the four donors are combined in the graph; each black circle represents a single cell (DMSO, n=94; A83–01, n=78). **(D)** Same as in C for cells immunostained with an antibody against Lamin A/C and counterstained with DAPI. Nuclei were segmented using ImageJ software and the nucleus size was calculated (DMSO, n=82; A83–01, n=91). The percentage of cells with micronuclei was determined by staining fixed cells dropped on glass slides with Giemsa and determining the number of nuclei with associated micronuclei as a percentage of the total number of nuclei imaged (DMSO, n=549; A83–01, n=528). **(E)** p21 and p16 were analyzed by qPCR using cDNA produced from basal epithelial cells at P3 treated with A83–01 or DMSO. Representative images of basal epithelial cells treated with A83–01 or DMSO immunostained with antibodies against p16 and p21, and counterstained with DAPI. **(F)** Representative images of basal epithelial cells cultured in MEGM or MEGM containing 500 nM A83–01 were immunostained with antibodies against p63 and cytokeratin 14 (CK14), followed by counterstaining with DAPI. Western blot analysis of cell lysates from these populations was performed using antibodies against CK14, p63, SMA, and Actin (loading control).

The application of A83–01, a potent inhibitor of TGF-β type I receptor ALK5 kinase, type I activin/nodal receptor ALK4, and type I nodal receptor ALK7, was sufficient to prevent stasis in basal epithelial cells (Fig. 3A). A83–01 inhibited EMT at the transcriptional level, as evidenced by the significantly increased expression of P-cadherin and the decreased expression of N-cadherin, Snail, Twist, and Zeb (Fig. 3B). TGF-β inhibition was associated with a highly significant decrease in cell size (Fig. 3C), reduction in nuclear area, and elimination of micronuclei (Fig. 3D). A83–01 treatment also significantly reduced p21 and p16 levels (Fig. 3E). We observed that treatment with A83–01 maintained the protein expression of p63, an important transcriptional regulator of basal cell differentiation (Fig. 3F). Together, these results implicate the engagement of the TGF-β pathway in cellular dysfunction and the activation of tumor suppressor pathways during stasis.

**Figure 4:**
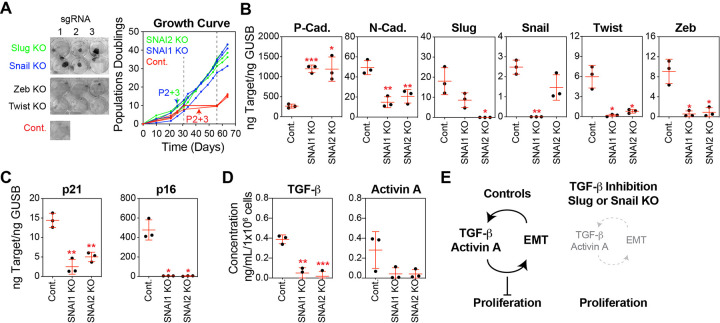
Depletion of EMT Transcription Factors Prevents Stasis in Basal Epithelial Cells. **(A)** Basal epithelial cells (P1) were transduced with lentiviral CRISPR constructs, containing CAS9 and sgRNA targeting Slug, Snail, Zeb, Twsit, or non-targeting controls (Cont.). These constructs also included an Internal Ribosome Entry Site (IRES)-GFP sequence, facilitating the selection of cells incorporating the vector using FACS. Snail knockout (KO), Slug KO, and control cells were expanded in MEGM, cells were counted at each passage, and a growth curve of the cumulative population doubling over time was plotted. Cells and CM were collected at P2+3 (P2 indicates that the transduced cells were at passage 2, and +3 indicates the number of passages following GFP^+^ selection), as indicated by the arrows. **(B)** P-cadherin, N-cadherin, Slug, Snail, Twist and Zeb were analyzed by qPCR using cDNA produced from basal epithelial cells at P2+3, selected for integration of Snail KO, Slug KO, or the control vector. **(C)** p21 and p16 were analyzed as described in B. **(D)** Sandwich ELISA kits were used to determine the concentrations of TGF-β and activin A. An equal number of cells were harvested from basal epithelial cells at P2+3 selected for integration of Snail KO, Slug KO, or control vector, and cultured in basal media (supplements excluded) for 24 hours. Concentrations were normalized to the final cell number. **(E)** Graphic illustration of the key findings presented in Figs. 3 and 4. In control basal epithelial cell cultures in serum-free MEGM, positive feedback between activation of the TGF-β pathway and induction of EMT generates an environment that suppresses proliferation, causing stasis. In basal epithelial cell cultures treated with a TGF-β inhibitor or transduced with lentiviral CRISPR vectors targeting Snail or Slug, the levels of TGF-β pathway activation and EMT induction, as well as the positive feedback loop between them, were diminished, permitting proliferation and avoiding stasis.

Similar to the treatment of basal epithelial cells with A83–01, we found that CRISPR-mediated knockout (KO) of Snail or Slug eliminated the stasis barrier compared with non-targeting controls (Fig. 4A). Following selection, Snail and Slug KO basal epithelial cells generated tight colony morphologies similar to those produced by the variant basal epithelial cultures emerging from stasis, whereas non-targeting controls were more dispersed across the culture surface. In contrast, Zeb and Twist KO basal epithelial cells demonstrated a growth pattern similar to that of the non-targeting control, and were therefore not followed up in culture (Fig. 4A). Targeting Snail and Slug using CRISPR vectors reduced their transcript levels. Consistent with their role in the EMT network, Snail and Slug knockout significantly increased the transcript levels of P-cadherin and decreased those of N-cadherin, Twist, and Zeb compared to non-targeting controls (Fig. 4B). Basal epithelial cells with Snail or Slug KO demonstrated substantially lower transcription of p21 and p16 tumor suppressors than the controls (Fig. 4C). We observed vimentin in the cytoskeleton of basal epithelial cells expressing p16, consistent with the idea that EMT is correlated with the senescence phenotype in this culture system (Fig. S2A). Notably, the reduced growth arrest observed upon Snail and Slug KO was associated with decreased levels of TGF-β and the TGF-β superfamily member activin A (Fig. 4D), which are part of the autocrine and paracrine loops that promote the EMT program[54] and perpetuate senescence[49].

In contrast, overexpression (OE) of the EMT-related transcription factors Snail and Slug immediately suppressed the growth of basal epithelial cells, preventing their expansion (Fig. S2B). In this experiment, Snail and Slug were expressed at non-physiological levels, which were far higher than those observed in basal epithelial cells at stasis. Analysis of these cultures at the first passage showed a high degree of EMT, as evidenced by the downregulation of P-cadherin and upregulation of N-cadherin, Twist, and Zeb (Fig. S2C). Further examination of this model was not feasible because of the rapid and complete cessation of cell growth.

In summary, TGF-β pathway inhibition or Snail or Slug knockdown prevented EMT activation and stasis in basal epithelial cells. Activation of the TGF-β pathway induces the expression of EMT-related transcription factors, such as Snail, Slug, Zeb, and TWIST, which promote the transition from an epithelial to a mesenchymal phenotype. In turn, mesenchymal cells resulting from EMT secrete additional TGF-β and Activin A creating a positive feedback loop that sustains and amplifies the EMT process. The activation of TGF-β and EMT in basal epithelial cells leads to growth suppression and cellular dysfunction, as is evident in conventional culture systems (Fig. 4E).

#### EMT Generates a Spectrum of Basal Epithelial Cell States with Varying Proliferative and Self-Renewal Capacities

**Figure 5:**
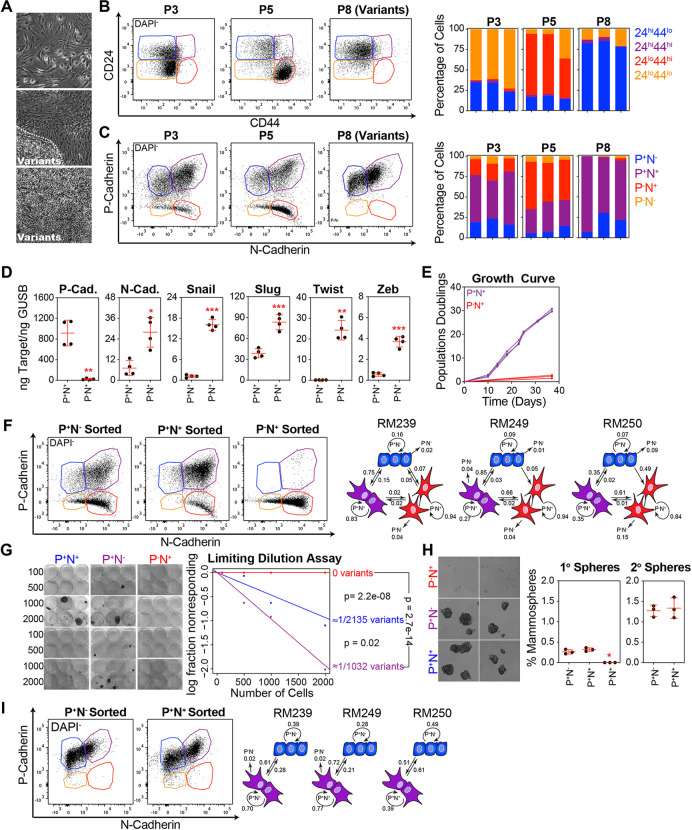
EMT Generates a Spectrum of Basal Epithelial Cell States with Varying Proliferative and Self-Renewal Capacities. **(A)** Phase contrast images of P5 basal epithelial cells demonstrating different morphologies. **(B)** Dead cells (DAPI^+^) and doublets were removed, and CD44 and CD24 were visualized using flow cytometry. The CD44^hi^CD24^lo^, CD44^hi^CD24^hi^, CD44^lo^CD24^hi^, and CD44^lo^CD24^lo^ immunophenotypes were quantified. Each bar represents the basal epithelial cells isolated from a single donor. **(C)** Same as B, using antibodies against P-cadherin and N-cadherin. Immunophenotypes were gated based on singly stained controls as P^−^N^−^, P^+^N^−^, P^+^N^+^, and P^−^N^+^. **(D)** P-cadherin, N-cadherin, Snail, Slug, Twist, and Zeb were analyzed by qPCR using cDNA produced from basal epithelial cells (P5) directly sorted using FACS based on a P^+^N^−^ or P^+^N^+^ immunophenotype. **(E)** P^+^N^−^ or P^+^N^+^ basal epithelial cells (P5) were expanded in MEGM and counted at each passage. **(F)** P^+^N^−^, P^+^N^+^, and P^−^N^+^ basal epithelial cells (P5) were sorted into 6-well plates, cultured for 14 days in MEGM, and re-analyzed by flow cytometry. The illustration on the right shows the proportion of cells that transitioned between states in three donors: P^+^N^−^ in blue, P^+^N^+^ in purple, and P^−^N^+^ in red. **(G)** P^+^N^−^, P^+^N^+^, and P^−^N^+^ basal epithelial cells (P5) were sorted into 6-well plates at concentrations of 100, 500, 1000, and 2000 cells and cultured in MEGM until variant colonies were observed. Estimates of variant frequency in the sorted populations and p-values were determined by Extreme Limiting Dilution Analysis [84] using the online resource: https://bioinf.wehi.edu.au/software/elda/. **(H)** P^+^N^−^, P^+^N^+^, and P^−^N^+^ basal epithelial cells (P5) were sorted into ultra-low-adherence plates containing mammosphere medium [44] (DMEM/F12 supplemented with B27, EGF, FGF2, and heparin). The mammospheres were visualized after 14 days and counted using a phase-contrast microscope. **(I)** P^+^N^−^ and P^−^N^+^ cells were sorted from P8 (variant) basal epithelial cell cultures and re-analyzed by flow cytometry after 14 days. The illustration on the right shows the proportion of cells that transitioned between states in three donors: P^+^N^−^ in blue and P^+^N^+^ in purple. Note that very few P^−^N^+^ cells were observed in variant cultures (see panel C).

Limiting Activation of the TGF-β pathway and EMT induction was sufficient to avoid stasis in the entire cultured basal epithelial cell population. However, rare basal epithelial variants were intrinsically capable of avoiding stasis. Variants can be visualized as epithelial colonies (tightly clustered and adhered to their neighbors) emerging among cells with spindle or senescent-like morphologies (Fig. 5A). EMT is a dynamic process that enables epithelial cells to acquire mesenchymal properties, leading to increased plasticity and the ability to transition between multiple cellular states [21–26]. This process creates a spectrum of intermediate phenotypes that allow cells to exhibit varying degrees of epithelial and mesenchymal characteristics.

Many studies have used CD24 and CD44 to distinguish between epithelial and mesenchymal states [25]. Indeed, almost all cells in stasis cultures expressed the CD44^hi^CD24^lo^ phenotype, consistent with the high levels of EMT at this time point. In contrast, pre-stasis HMECs and post-stasis variants mostly lacked CD44 (Fig. 5B). However, this method was unable to separate epithelial, intermediate, and more complete mesenchymal phenotypes. To better distinguish between these EMT states in basal epithelial cell cultures, we developed an alternative method that utilized antibodies against P-cadherin, an epithelial marker highly expressed by basal epithelial cells, and N-cadherin, a marker highly expressed in mesenchymal cell types. In early and stasis cultures, we observed complete EMT states expressing N-cadherin but lacking P-cadherin (P^−^N^+^), an intermediate EMT state expressing both P- and N-cadherins (P^+^N^+^), and an epithelial state expressing P-cadherin but lacking N-cadherin (P^+^N^−^). In cultures dominated by variants, the complete EMT state, P^−^N^+^, was mostly absent (Fig. 5C). We sorted basal epithelial cells in the P^+^N^+^ and P^−^N^+^ states from stasis cultures (P5). At the mRNA level, P^−^N^+^ cells exhibited significantly lower levels of P-cadherin and higher levels of N-cadherin, Snail, Slug, Twist, and Zeb than did P^+^N^+^ cells, confirming that P^−^N^+^ populations represent a higher degree of EMT (Fig. 5D). Notably, basal epithelial cells with a complete EMT phenotype were growth arrested and could not be passaged, although they remained adherent to the culture surface for several weeks (Fig. 5E).

Early passage basal epithelial cells were sorted based on the immunophenotypes P^+^N^−^, P^+^N^+^, and P^−^N^+^) corresponding to different states along the EMT spectrum, and cultured for 14 days. When the resulting populations were re-analyzed, both P^+^N^−^ (blue) and P^+^N^+^ (purple) cells were able to recapitulate the entire spectrum of EMT states, whereas P^−^N^+^ (red) cells were restricted to the ‘fully’ mesenchymal state (Fig. 5F). When separated from stasis cultures before the emergence of variants, we demonstrated, using limiting dilution analysis, that basal epithelial cells in the P^+^N^−^ and P^+^N^+^ states could give rise to variant colonies, whereas cells in the P^−^N^+^ state could not (Fig. 5G). In addition to the ability to form variant colonies in monolayer culture, we observed that basal epithelial cells in the P^+^N^−^ and P^+^N^+^ states could form mammospheres under non-adherent conditions, which is suggestive of self-renewal and stem cell properties [44]. In contrast, cells in the P^−^N^+^ state were unable to create mammospheres (Fig. 5H). The P^−^N^+^ state was completely absent in the variant cultures, and cells could only move between the P^+^N ^−^ and P^+^N^+^ states, suggesting attenuation of the EMT program (Fig. 5I).

In summary, isolated basal epithelial cells initially activated an EMT program that could progress to a fully mesenchymal state, ultimately leading to growth arrest. However, over several passages, we observed stabilization of the epithelial and intermediate EMT states, restricting access to the fully mesenchymal state. This restriction was associated with the emergence of variants that retained plasticity and could transition between epithelial and intermediate EMT states but not the fully mesenchymal state.

#### PRC2-Mediated Epigenetic Regulation Governs Access to EMT States in Basal Epithelial Variants

**Figure 6:**
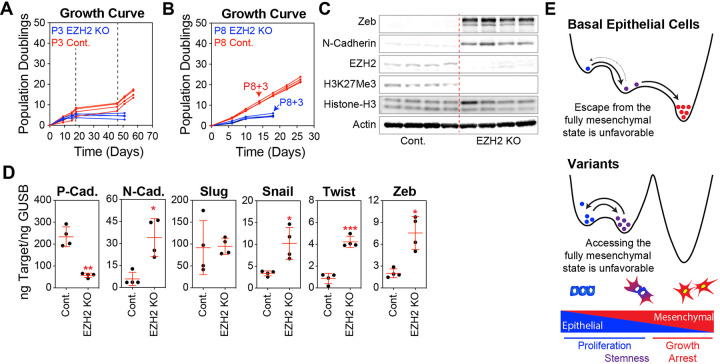
PRC2-Mediated Epigenetic Regulation Governs Access to EMT States in Basal Epithelial Variants. **(A)** Sorted basal epithelial cells (P3) were transduced with lentiviral CRISPR constructs containing CAS9 and sgRNA targeting EZH2 or non-targeting controls (Cont.). These constructs included an IRES-GFP sequence, which facilitated the selection of cells incorporating the vector using FACS. EZH2 and control cells were expanded in MEGM; at each passage, cells were counted, and a growth curve of the cumulative population doubling over time was plotted. **(B)** Same as in A, starting with P8 (variant) basal epithelial cells. Cells were collected at P8+3, as indicated by the arrows. **(C)**Western blot analysis of lysates from P8 EZH2 KO and control cells using antibodies against Zeb, N-cadherin, EZH2, H3K27me3, total Histone H3, and Actin ( loading control). **(D)** P-cadherin, N-cadherin, Slug, Snail, Twist, and Zeb were analyzed by qPCR using P8+3 EZH2 KO and control cells. **(E)** In the EMT continuum, cells exist in various states ranging from epithelial to mesenchymal phenotypes, including metastable intermediate transition states. Epigenetic processes involving PRC2 and likely other mechanisms are altered during adaptation to unfavorable cell culture conditions. These alterations influence the transitions between states and contribute to the stabilization of cells within intermediate EMT states.

PRC2 is known to play a critical role in epigenetic regulation of gene expression in variant basal epithelial cells. Indeed, by catalyzing the trimethylation of histone H3 on lysine 27 (H3K27me3), PRC2 silences the target genes involved in growth and differentiation. Over time in culture, silencing of many PRC2 targets can be reinforced by promoter hypermethylation [35, 41]. EZH2, the catalytic subunit of PRC2, is central to this function, and its knockout disrupts the ability of this complex to regulate gene expression. We found that the knockout of EZH2 in basal epithelial cells at P3 reliably prevented the formation of variant colonies, which was expected given that PRC2 plays a well-established role in silencing CDKN2A and removing other obstacles to proliferation (Fig. 6A). EZH2 knockout in the P8 variant basal epithelial cells also impaired cell proliferation (Fig. 6B).

Recent studies have revealed that PRC2 plays a crucial role in controlling the EMT trajectory by repressing specific genes involved in EMT [56]. In EZH2 knockout basal epithelial variants, we confirmed a decrease in EZH2 expression and global H3K27me3 levels at the protein level, and observed increased levels of Zeb and N-cadherin (Fig. 6C). EZH2 knockout promoted EMT at the transcriptional level, as evidenced by decreased P-cadherin, but increased N-cadherin, Snail, Twist, and Zeb expression (Fig. 6D).

By reducing the activation of the EMT program, PRC2 and potentially other epigenetic regulators maintain variant basal epithelial cells in an intermediate EMT state that favors continued proliferation and stemness, and disfavors the path to complete EMT states associated with growth arrest (Fig. 6E).

#### TGF-β Inhibition Prior to Cellular Transformation Reduced Metaplastic Phenotypes in Basal Epithelial-Derived Tumors

**Figure 7:**
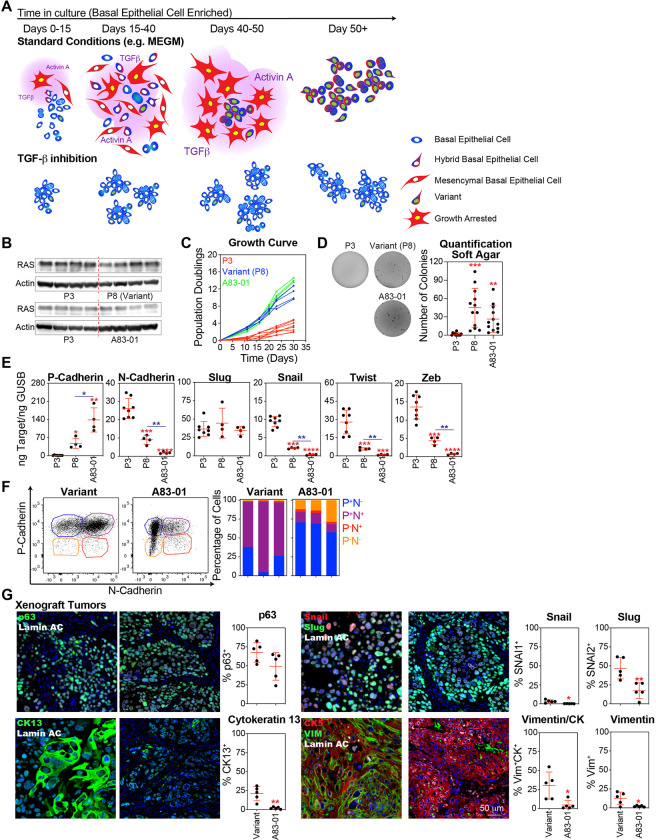
Inhibition of the TGF-β pathway during cellular transformation reduces metaplastic phenotypes in basal epithelial-derived tumors. **(A)** Activation of the TGF-β pathway and EMT collaborate to drive cellular dysfunction and growth arrest. Adapted variants that emerged from stasis cultures exhibit notable changes in gene expression associated with growth and differentiation and are stabilized in an intermediate EMT state. Inhibition of the TGF-β pathway eliminates these selective pressures during the expansion of basal epithelial cells. **(B)** Basal epithelial cell populations: (1) P3, cultured in MEGM + DMSO for three passages, (2) variant, cultured in MEGM + DMSO for eight passages, and (3) A83–01, cultured in MEGM + 500 nM A83–01 for three passages, were oncogenically transformed through sequential transduction with TERT and SV40, followed by selection (puromycin and hygromycin) and lentiviral transduction of oncogenic KRAS^G12V^ followed by a second round of selection (blasticidin and IRES-RFP). Western blot analysis was performed on lysates from these oncogenically transformed basal epithelial cells using antibodies against RAS and Actin (as a loading control). **(C)** At each passage, Oncogenically transformed basal epithelial cells were counted. A growth curve of the cumulative population doubling over time was plotted. **(D)** Transformed basal epithelial populations were suspended in soft agar in each well of a 6-well plate at a concentration of 20,000 cells/well and cultured in DMEM/F12 + 20% FBS. After four weeks, the resulting colonies were stained with crystal violet, imaged, and counted using the ImageJ software. **(E)** P-cadherin, N-cadherin, Slug, Snail, Twist, and Zeb were analyzed qPCR using cDNA produced from oncogenically transformed basal epithelial cell populations. **(F)** Variant and A83–01-treated oncogenically transformed basal epithelial cells were immunostained for P-cadherin and N-cadherin, then visualized by flow cytometry. **(G)** Variants and A83–01-treated oncogenically transformed basal epithelial cells (4 × 10^6^) were injected subcutaneously with 50% Matrigel into 8-to 12-week-old female NSG mice. Tumors formed over a period of 3–6 months. FFPE sections from five tumors were subjected to multiplexed immunohistochemical analysis for p63, cytokeratin 13 (CK13), Slug + Snail, and cytokeratin 5 + vimentin. Human-specific lamin A/C was used to identify the tumor cells.

Basal epithelial progenitors have been proposed as the cells of origin for MBC [9], a rare histological subtype associated with elevated levels of EMT compared with other breast cancer subtypes [18] (Fig. S3A and S3B). In culture, basal epithelial cells strongly activated the TGF-β pathway and induced EMT. The selection of variants was associated with the stabilization of intermediate EMT states capable of continued proliferation, limiting access to a complete mesenchymal state subject to growth arrest. Pharmacological inhibition of the TGF-β pathway suppressed the activation of the EMT program and eliminated the selective pressure that promoted the outgrowth of the basal variants (Fig. 7A).

To better understand the role of TGF-β and EMT-dependent selection of the variant phenotype in MBC carcinogenesis, we employed an established transformation protocol [9] involving the sequential transduction of TERT, SV40 T antigen, and mutant K-Ras^G12V^. We compared the oncogenic transformation of P3 basal epithelial cell populations cultured in MEGM for three passages, poised to enter stasis, with variant basal epithelial cell populations cultured in MEGM for eight passages that had already overcome stasis. Additionally, we compared P3 basal epithelial cell populations cultured in MEGM + DMSO with those cultured in MEGM + A83–01 for three passages, which suppressed EMT and allowed them to avoid stasis and variant selection. Notably, both the oncogenically transformed variant and A83–01 basal epithelial populations expressed levels of the mutant K-Ras^G12V^ equivalent to those of the P3 controls (Fig. 7B). Proliferation of oncogenically transformed P3 basal epithelial cells was suppressed, whereas the oncogenically transformed variant and A83–01 basal epithelial populations exhibited exponential growth kinetics (Fig. 7C). Additionally, oncogenically transformed P3 basal epithelial cells formed dramatically fewer colonies in soft agar, with most replicates showing no colony formation at all, indicating a reduced capacity for anchorage-independent growth compared to the variant and A83–01 basal epithelial populations, which showed no significant difference in their ability to form colonies in soft agar (Fig. 7D). A greater level of EMT activation was observed in oncogenically transformed P3 basal epithelial cells compared to both the variant and A83–01 populations, as evidenced by the lower levels of P-cadherin and higher levels of N-cadherin, Snail, Twist, and Zeb compared to the variant and A83–01 basal epithelial cell populations. In contrast, transformed variant populations exhibited intermediate levels of EMT, characterized by significantly higher levels of P-cadherin and lower levels of N-cadherin, Snail, Twist, and Zeb, compared to A83–01-treated basal epithelial populations (Fig. 7E). Flow cytometric analysis of P-cadherin and N-cadherin expression confirmed that oncogenically transformed variant populations predominantly exhibited intermediate EMT phenotypes (P^+^N^+^). In contrast, oncogenically transformed basal epithelial cells treated with A83–01 mostly expressed P-cadherin, with relatively little N-cadherin expression, thus mostly preserving their epithelial state (Fig. 7F).

Only the oncogeneically transformed variant and A83–01 basal epithelial cell populations formed tumors when xenotransplanted subcutaneously into NSG mice (Fig. S3C). Tumors derived from both A83–01-treated and variant basal epithelial cells were highly proliferative based on the number of cells that stained positive for Ki67 (Fig. S3D). Both sets of tumors expressed p63, which is a typical phenotype of MBCs, and several even rarer histological subtypes likely arising from basal epithelial progenitors [57–59]. Notably, A83–01 tumors exhibited a lower frequency of cells expressing the squamous differentiation marker cytokeratin 13, the EMT markers Snail and Slug, and the mesenchymal differentiation marker vimentin when compared to variant tumors (Fig. 7G).

In summary, high levels of EMT in the malignantly transformed P3 basal epithelial group were associated with poor growth and tumorigenicity, whereas the variant basal epithelial group, which exhibited intermediate levels of EMT gene expression, demonstrated enhanced growth and tumorigenicity. Inhibition of the TGF-β pathway, which significantly reduced EMT gene expression, promoted growth and tumorigenicity in the P3 basal epithelial group but led to a loss of metaplastic squamous and metaplastic elements.

## Discussion

In conventional monolayer cultures [29], HMECs are deprived of instructive biophysical and biochemical cues that maintain cellular homeostasis and cooperation within tissues. Instead, they form attachments with a non-compliant plastic substratum and proliferate in response to signals presented in an engineered medium. Consequently, HMECs enter stasis, the first, ‘stress-associated’ barrier to indefinite proliferation, during which most cells adopt a senescence-like phenotype [8, 33, 34]. To address this limitation, more recently developed culture systems optimized to support epithelial proliferation incorporate fibroblast feeders or hydrogels containing basement membrane components, mimicking key aspects of the *in vivo* microenvironment lost in conventional monolayer cultures. These systems also employ chemical inhibitors to reduce the ability of the cells to engage in stress response pathways. Collectively, these modifications allow cultured breast epithelial cells to avoid both stasis and agonescence, the second barrier to indefinite proliferation resulting from telomere attrition, without the need for genetic manipulation. Notably, these culture systems support genetic and epigenetic stability during long-term propagation [60, 61].

In such conventional monolayer cultures, rare variants activate an aberrant yet remarkably consistent epigenetic program that enables them to adapt to unfavorable culture conditions and reinitiate proliferation after encountering the stasis barrier [35–37]. Many epigenetic alterations favored in variant cultures, including hypermethylation of the CDKN2A promoter, have also been observed during human tumor progression [35]. In intact tissues, etiological factors, such as aging, chronic inflammation, and environmental exposure, gradually disrupt homeostatic interactions. This progressive disruption contributes to a decline in the quality of the tissue microenvironments, which is hypothesized to favor the selection of clones (epigenetic progenitors) that are adaptive to altered microenvironments, potentially leading to the emergence of cancer over the course of many decades [62, 63]. Arguably, conventional monolayer cultures represent an analogous process, albeit with a significantly accelerated time scale. Thus, variants within these cultures have proven useful in elucidating the mechanisms underlying the emergence of cancer progenitor states [25, 26].

In this study, we report that robust activation of the TGF-β pathway and induction of the EMT program are drivers of stasis and, therefore, constitute an important selective pressure underlying the emergence of the variant phenotype. Inhibition of the TGFβ pathway allowed basal epithelial cells to bypass stasis and dramatically reduced cell size, chromosomal instability, and expression of both p21 and p16. TGF-β plays a direct role in enhancing cell size by activating the mammalian target of rapamycin (mTOR) [64]. Notably, increased cell size is not simply a consequence of cellular senescence, but also a contributing factor through mechanisms that include failure to scale macromolecule production with cell volume causing cytoplasmic dilution, altered nuclear organization, chromosomal missegregation, DNA damage, and mitochondrial dysfunction [65–69]. TGF-β induces cellular senescence through SMADs, which can upregulate p15 and p21 and contribute to the establishment of SASP, which can reinforce senescence in an autocrine manner or induce senescence in neighboring cells in a paracrine manner [49–51]. Knockout of the key EMT factors Snail and Slug also allowed basal epithelial cells to bypass stasis. The EMT program is associated with increased activation of the TGFβ pathway through autocrine and paracrine secretion [54] but can also have negative consequences through other mechanisms. Indeed, during EMT, if cells continue to proliferate, they exhibit mitotic abnormalities such as centrosome clustering, cytokinesis failure, and chromosome missegregation, which result in increased DNA damage, aneuploidy, binucleated cells, and micronuclei. These mitotic defects are associated with the suppression of nuclear envelope proteins like LaminB1, critical for mitotic regulation [70, 71]. Thus, TGFβ pathway activation and EMT are key components of the stasis barrier, resulting in cellular arrest and dysfunction.

Notably, EMT is not a binary switch between epithelial and mesenchymal states but generates a spectrum of intermediates with both epithelial and mesenchymal traits. When basal epithelial cells underwent a complete transition to the mesenchymal state, which peaked at stasis, they were subject to proliferative arrest. In contrast, the re-initiation of proliferation within stasis cultures by variants was associated with the stabilization of intermediate states. These hybrid states are characterized by enhanced cellular plasticity and adaptability, which can lead to increased stem cell properties and tumorigenic potential [21–26]. PRC2 is known to contribute to the epigenetic program underlying the emergence of the variant phenotype by silencing genes involved in growth control and differentiation [35, 37, 41]. PRC2 also suppresses the expression of EMT-inducing transcription factors, such as ZEB1 and ZEB2 [56]. Indeed, in variant basal epithelial cells, knockout of EZH2, the catalytic subunit of PRC2, increased EMT gene expression, influencing the trajectory of the EMT program. Significantly, epigenetic alterations in the TGF-β pathway have been previously observed in variants [35, 38, 72]. EZH2 knockout in basal epithelial cells and variants impaired PRC2 function and diminished basal epithelial proliferation, limiting our study. In a prior study, fully transformed HMLER cells, which were derived from variant cultures, were not subject to growth arrest in response to compromised PRC2 activity; instead, they transitioned into a quasi-mesenchymal state associated with high metastatic potential. In contrast, the loss of another epigenetic regulator, KMT2D, results in a highly mesenchymal state [56]. Further research is warranted to elucidate the interplay between epigenetic regulators and EMT during the emergence of the variant phenotype. Nevertheless, our findings suggest that the adaptation of EMT toward favorable intermediate EMT states is a consequence of the epigenetic program, which facilitates the emergence of variants by modulating gene expression involved in growth control, differentiation, and other hallmark programs characteristic of a cancer progenitor state.

Consistent with the idea that variants are representative of a cancer progenitor state, the variant state confers tolerance to oncogenic insults such as RAS activation, thereby bypassing oncogene-induced senescence [39]. In this study, we also observed the increased vulnerability of variants to oncogenic transformation when compared to pre-stasis basal epithelial cells, which had not undergone the same epigenetic reprogramming characteristic of the variant population. TGF-β inhibition, which bypassed stasis in basal epithelial cultures, improved the transformation efficiency of pre-stasis basal epithelial cells, suggesting that overcoming the cellular arrest and dysfunction resulting from activation of the TGFβ pathway and EMT is necessary for achieving malignant transformation in these cells. This result was consistent with a previous report that found that either a dominant-negative TGFβ type II receptor or a TGFβ pathway inhibitor was able to bypass RAS-induced growth arrest variants and HMECs with compromised p16/Rb and p53. This study reported no difference in oncogenic transformation efficiency between pre-stasis HMEC and variant HMEC populations [73]. However, they compared pre-stasis HMEC (specimen 48R, batch T) with post-selection HMEC (specimen 48R, batch S), which were developed in different media systems—serum-free MCDB170 medium (a non-commercially available version of MEGM) versus serum-containing M87A supplemented with cholera toxin and oxytocin [74], which differed from our analysis of basal epithelial cells cultured in MEGM. Strikingly, the oncogenic transformation of basal epithelial cells resulted in metaplastic tumors with squamous and mesenchymal components in immunocompromised mice, which is consistent with previous reports [8, 9]. However, treatment with TGF-β pathway inhibitors generated tumors with a marked reduction in metaplastic elements, even though the TGF-β pathway inhibitor was not maintained in culture after selection for KRAS^G12V^ or in mice.

Basal cells adapt to conventional HMEC culture systems by restricting the EMT program to a hybrid epithelial/mesenchymal state that maintains a high proliferation, stemness, and tumorigenicity. This cellular plasticity is required to achieve a basal progenitor state that gives rise to rare MBCs. Notably, the MBC-like tumor phenotypes observed in the variant tumors show similarities to squamous cell carcinomas originating from basal epithelial cells in stratified epithelia throughout the body, such as lung, head and neck, esophageal, and cutaneous squamous cell carcinomas [75]. A subset of these squamous tumors are associated with mesenchymal differentiation, akin to that sometimes observed in MBCs [17, 18], often referred to as spindle cells or sarcomatous elements [76]. Both MBC and squamous cell carcinoma are associated with inactivation of CDKN2A [77–81]. Chronic inflammation is often a contributory factor in the development of squamous cell carcinoma, often linked to tobacco use in lung, head, neck, and esophageal cancers, as well as to ultraviolet radiation in cutaneous squamous cell carcinomas [75]. MBC has been observed in the clinical context of chronic inflammation, such as breast abscess associated with a recurrent or long-standing breast cyst or implant capsule [82]. Evidence of intermediate EMT states has been identified in single-cell RNA-sequencing and immunohistochemical studies of squamous cell carcinomas, which are hypothesized to play a role in promoting aggressive tumor properties, including the formation of circulating tumor cell clusters that are highly metastatic and exhibit cancer stem cell characteristics [83].

Variants and their derivatives have been widely used to model breast epithelial cell biology and carcinogenesis, facilitating many influential observations. However, it is essential to note that these cultures selectively favor basal epithelial cells, which are thought to be the origin of a small fraction of breast cancers, mainly MBCs [9]. Despite this limitation, findings from HMEC culture systems are often extrapolated to breast cancer as a whole, although they may not accurately represent the biology of the more common breast cancer subtypes.

## Conclusions

This study illuminated dynamic processes operative in the HMEC model system, which is widely used to study epithelial cell biology and carcinogenesis. Following disruption of their native microenvironment, activation of the TGF-β pathway and induction of EMT drove stasis in HMEC cultures, exerting selective pressure that favored the emergence of rare variant basal epithelial cells. The epigenetic stabilization of intermediate EMT states in variants contributed to their enhanced proliferative capacity and avoided complete mesenchymal differentiation, which was associated with irreversible growth arrest. The ability of basal epithelial cells to navigate the spectrum of EMT states and stabilize favorable intermediate states contributed to the emergence of a cancer progenitor state that gives rise to tumors with histopathological features consistent with rare MBCs and may overlap with tumor types initiated from basal epithelial cells, particularly squamous cell carcinomas, in other tissues.

## Data Availability

All data generated for this study are available in the paper and its supplementary information files.

## Abbreviations

HMEC: Human mammary epithelial cell
CDH1: cadherin1 (E-cadherin)
CDH2: cadherin2 (N-cadherin)
CDH3: cadherin 3 (P-cadherin)
CDKN2A: Cyclin-dependent kinase inhibitor 2A
CM: Conditioned medium
EMT: Epithelial-mesenchymal transition
EZH2: Enhancer of zeste homolog 2
H3K27me3: Trimethylation of lysine 27 on histone H3
MBC: Metaplastic breast cancer
MEGM: Mammary epithelial cell growth medium
PRC2: Polycomb repressive complex 2
SASP: Senescence-associated secretory phenotype
SEEP: Stress-elicited extrinsic phenotype
SNAI1: Snail family transcriptional repressor 1(Snail)
SNAI2: Snail family transcriptional repressor 2 (Slug)
TGF-β: Transforming growth factor-beta
TNBC: Triple-negative breast cancer
TWIST1: Twist family BHLH transcription factor 1 (Twist)
ZEB1: Zinc finger E-box binding homeobox 1 (Zeb)
